# Genomic, proteomic and lipidomic evaluation of endometrial receptivity

**DOI:** 10.4274/tjod.98475

**Published:** 2015-12-15

**Authors:** İrem Demiral, Murat Doğan, Ercan Baştu, Faruk Buyru

**Affiliations:** 1 İstanbul University Faculty of Medicine, Department of Obstetrics and Gynecology, İstanbul, Turkey; 2 Acıbadem Fulya Hospital, Division of Reproductive Endocrinology and Infertility, İstanbul, Turkey

**Keywords:** Endometrial receptivity, genomic, lipidomic, proteomic

## Abstract

Endometrial receptivity is a complex phenomenon that plays a vital role in infertility. Although quality of embryo can be evaluated for a successful implantation, endometrial receptivity is still an unknown factor. With advances in technology, the microarray approach has provided an ‘omic’ tool to evaluate endometrial receptivity. In Latin, ‘omic’ means the whole family. The genomic, proteomic, and lipidomic evaluations of endometrium mean a wholesome evaluation of the genes, lipids and proteins of the endometrium. Evaluation of receptivity with this three-way approach may provide insight to the potential markers of implantation. Genomic analysis has been limited to date because not every gene alteration affects protein expression. Lipidomic analysis has recently gained popularity because lipids are strictly controlled during the implantation period. In summary, with the recent advances in microarray technology, genomic, lipidomic, and proteomic analyses of the endometrium may provide ‘optimal’ evaluation tools and criteria to assess receptivity in the near future.

## INTRODUCTION

Three important events have to occur during the window of implantation in order to form a healthy pregnancy:^(1)^ proper embryo development^(2)^ development of a receptive endometrium, and^(3)^ a successful interaction between the embryo and endometrium. Even when optimal conditions were met, the pregnancy rate was found to be around 30%^(1)^.

The endometrium undergoes cyclic morphologic and molecular changes in preparation for pregnancy. Understanding these changes is important in order to clarify the basic biology of the uterus and the environment of the endometrial tissue that allows implantation. Recent studies showed that the uterine environment allows implantation and provides adequate nutrition to the embryo to ensure its development^(2,3)^.

Despite all the advances in reproductive medicine, in vitro fertilization (IVF) success rates are still low. It is thought that the reason of low pregnancy rates in IVF cycles is largely due to problems in endometrial receptivity. In order to access the endometrial receptivity, many parameters such as endometrial morphology or subendothelial blood flow have been used, but their prognostic values were found to be low^(4)^. For the last 20 years, regardless of the advances in embryo quality improvement and ‘best’ embryo selection, the progress to a better understanding of endometrial function has lagged behind the biology of the blastocyst. Even though the endometrium can be assessed in a non-invasive way thanks to the current imaging methods, endometrial receptivity is still based upon morphologic features, which has no clinical reflection^(5,6)^. As a result, alternative diagnostic methods to replace or accompany conventional methods are needed. Today, we know that the endometrium changes its normal structure during the IVF cycles as a result of ovarian stimulation^(7,8)^. The endometrium responds to exposure of high progesterone levels with the premature injection of human chorionic gonadotropin, used instead of luteinizing hormone (LH), by changing its constitution. When stimulated cycles were compared with normal cycles, a significant difference in cytokine levels were reported^(9,10)^. This is why markers that reflect endometrial receptivity are needed; patients will not be exposed to expensive IVF treatments and better success rates will be achieved.

With the recently increasing use of microarray technology,^(11)^ it is possible to analyze thousands of genes in one particular sample. By courtesy of this technology, we now have the opportunity to analyze the genomics of endometrial development. Additionally, in recent years, with the progression seen in the domain called ‘omics,’ extensive molecular comparison of cells and tissues has been made possible. With the help of this technologic evolution in reproductive medicine, the opportunity to reveal the molecular signature of many cells and organs related to reproduction has arrived. During the stages of the menstrual cycle, by analyzing different expression of genes in the endometrium with microarray, diagnostic markers that reveal the implantation period can be found. With the aid of proteomic analyzers, proteins that have effects on reproduction can be discovered. In this context, progress by definition has occurred in the ‘omics’ world: genomics defines the analysis of genes, ‘proteomics’ defines the analysis of proteins, and also functional classifications (such as secretomics, metabolomics, transcriptomics, epigenomics) have been made. In light of this new classification, secretomics is the global definition of factors secreted by cells or organs in physiologic or pathologic conditions^(12,13)^. If we look at the correlation with the uterus, endometrial fluid or tissue samples can be collected using minimal invasive methods and by doing so, endometrial receptivity can be analyzed.

The aim of this review was to focus on the potential advantages of investigating endometrial receptivity, an area that has been studied the least in reproductive medicine, and to scrutinize the clinical implications for IVF in view of current methods of analysis such as genetic expression (genomics), secretomics, proteomics, and lipidomics.

## ENDOMETRIAL ENVIRONMENT AND REPRODUCTION

In humans, the endometrium undergoes two phases during the menstrual cycle: the proliferative and secretory phases. In each phase, endometrial tissue has its own morphologic and physiologic features. In the middle of secretory phase, the endometrial secretions that fill the uterine lumen interact with the blastocyst. This is called the window of implantation. Special interest is given to these secretions for two reasons: the first is the molecular content of these secretions may be a marker for the endometrium to allow implantation. This situation could have a very important diagnostic value for IVF. The second revolves around the exact molecular environment when the blastocyst enters the uterine lumen; this would give us information about how the endometrium becomes receptive to implantation and amenable to blastocyst development. 

The role of the endometrial fluid was recently studied for its capacity to provide adequate nutrition to the developing embryo. This situation is crucial for implantation as much as for the subsequent embryo development. According to animal studies, changes in the mother’s diet can lead to adverse outcomes in infants^(14,15,16,17)^. Before implantation, it is understood that the embryo makes changes in order to adapt to the environment, cellular distribution between trophectoderm and inner cell mass, function of mitochondria, and cellular energy sensors and signal system sensitivity, each of which is associated with metabolism (amino acid cycle, carbohydrate metabolism, nutrition transport)^(14,18,19,20,21)^.

## ANALYSIS OF ENDOMETRIAL RECEPTIVITY

Endometrial receptivity is mostly dependent on the local effects of estrogen and progesterone^(22)^. These effects, through stimulation of the cellular and molecular responses of the endometrium, can be defined as the endometrial receptivity window, which occurs between the 19^th^ and 23^rd^ day of the menstrual cycle. For more than 50 years, the gold standard of clinical assessment of the endometrium has been the histologic criteria described by Noyes et al.^(23)^. These investigators described the specific morphologic appearance of the different parts of the endometrium during the menstrual cycle. The classic work of Noyes et al.^(23)^ has been cited thousands of times in the literature and has been accepted worldwide as the diagnostic tool in endometrial research. However, in the last ten years, some randomized studies reported that the histologic analysis per se may not be sufficient to predict receptivity^(5,6)^. The fact underlined by these studies was the need for new technologies that will enable objective analyses of endometrial samples and development.

Among the new technologies, microarray is undoubtedly the most used one in reproductive medicine. Ponnampalam et al.^(24)^ were the first investigators to reveal the genomic character of the endometrium during the menstrual cycle using microarray technology. These investigators reported that it was possible to stage the endometrium very acutely according to the transcriptional profile, independent of the morphologic appearance. Most importantly, by identifying characteristics of variant genes in different phases of the menstrual cycle, they showed that gene expression profiling was a potential instrument in the analysis of endometrial receptivity.

Some studies reported that in cycles where ovarian stimulation was performed, high estrogen and/or progesterone levels deteriorated endometrial receptivity^(25,26)^. High estrogen concentrations may lead to early endometrial secretion by previous stimulation of progesterone receptors in the endometrium. Researchers have reported that GnRH agonists and antagonists have little effect on receptivity compared with natural cycles,^(27,28)^ and others reported a profound impact^(29,30,31,32,33,34)^. Steps that require investigation in the future are the similarities or differences of the effects of GnRH agonists and antagonists on endometrial receptivity.

As an alternative to genomics analysis, proteomic and/or lipidomic analysis of uterine fluid and tissue samples can give precious information about molecular phsysiology^(35,36)^. During IVF, this kind of small-sample-size and quick-result technology can provide a sensitive and accurate assessment of endometrial receptivity through easy samples collection from the uterus via a minimally invasive approach. Research conducted in the last ten years has shown that fluid aspiration performed before embryo transfer can be performed with no adverse effect on pregnancy rates in IVF cycles^(37,38)^.

## GENOMIC ANALYSIS OF THE ENDOMETRIUM

With the development of new microarray technologies, many investigators have started genomic analysis in reproductive medicine^(39,40,41,42,43)^. Many important molecules have been identified with these studies: genes responsible for lipid metabolism (apolipoprotein D), immune response (decay accelerating factor-CD55, serine or cysteine proteinase, interleukin (IL)-15), control of the cell cycle (cell growth arrest and TNF-α-induced DNA damage), or enzymes whose function differs in different tissues (monoamine oxidase). There are many studies in the literature about the changes in the endometrium’s gene expression profile during the transition from the pre-receptive- (LH+1/5) to the receptive period (LH+7/9)^(39,44,45,46,47)^. The number of genes whose expression differs between these two periods varies from 107 to 2878.

These genes, which might be the specific markers of endometrial receptivity, were identified in normal menstrual cycles. It is important to know if these genes have clinical value in predicting pregnancy in stimulated cycles. In a recently published study, the endometrial gene expression profile was analyzed in patients for whom ICSI treatment was planned at LH+7/9, 1-2 cycles before starting GnRH analogs. In patients who became pregnant, only 6 genes were found to have increased expression in the samples: vascular endothelial growth factor A (VEGFA), phospholipase A2 group 2A (PLA2G2A), alkaline phosphatase (ALP), leukemia inhibitory factor (LIF), nicotinamide N-methyltransferase (NNMT) and stanniocalcin 1 (STC1)^(48)^. Extensive cohort studies are needed to identify the predictive value of these genes for pregnancy outcomes.

The changes of gene expression seen in the endometrium does not always result in changes in proteins. The genomic analysis of the endometrium is insufficient to understand and identify receptivity in correlation with secreted proteins and other agents; this led to a focus on proteomic and lipidomic analysis. An interesting result of genomic analysis was the revealing of continuous control on lipid metabolism during endometrial development and the implantation window^(40)^. In this context, the importance of lipidomic analysis of endometrial tissue and fluids is underlined.

## PROTEOMIC ANALYSIS OF THE ENDOMETRIUM

As stated before, there is a significant increase in the protein content of fluids that fill the uterine lumen during the implantation window. As such, the explicit protein profile of uterine fluid in fertile women may play an important role in identifying the receptive endometrium. Proteomic analysis may help to predict the right implantation window or to determine how much of the endometrium is ‘open’ to receptivity during IVF approaches^(49,50,51,52,53,54)^. One common protein has been reported in studies where proteomic analysis of the endometrium was performed from tissue samples: annexin A4^(44,46)^. As previously discussed, the genomic changes do not always correlate with proteomic changes. For this reason, the results of six transcriptomic^(39,42,43,44,45,46,47)^ and two protemic^(44,46)^ studies conducted during the transition of the endometrium from the pre-receptive phase to the receptive phase were compared. Only gene of one protein (annexin A4) was found to be up-regulated in five transcriptomic and both proteomic studies^(42,43,44,45,46,47)^. This protein is thought to play a role in shaping the endometrial apical pole for cellular adhesion. Annexin A2 (ANXA2) and monoamine oxidase A (MAOA) genes were found over-expressed in three transcriptomic studies: ANXA2^(42,43,47)^, MAOA^(42,45,46)^ and one proteomic study^(44)^. It is shown in the literature that patients who experience implantation failures have decreased MAOA mRNA and protein expression^(55,56)^. Transgelin 2 (TAGLN, TGF-β/Smad-dependent epithelial cell migration) and L-plastin (LCP1, a member of the actine-binding protein family) were found to be over-expressed in two transcriptomic studies: TAGLN^(42,46)^, LCP1^(43,47)^ and one proteomic study^(44)^. From all these comparisons, information about five more genes was obtained; progesterone-receptor-membrane component 1 (PGRMC 1) and stathmin 1 (STMN) was found down-regulated in one transcriptomic study and this finding was supported in a proteomic study: PGRMC 1^(44,47)^, STMN^(43,44)^. Apolipoprotein L2 (APOL2), an aldehyde dehydrogenase 1 family member, A3 (ALDH1A3), and S100-calcium-binding protein A10 (S100A10) were found to be up-regulated in a transcriptomic study, and a proteomic study supported this finding^(44,46)^. The function of these five genes is as yet undiscovered.

There are some advantages in performing proteomic analysis in samples taken from endometrial fluid compared with tissue biopsy: the lower content of cellular protein in endometrial fluid makes the analysis more detailed and facilitates the identification of proteins and low-concentration post-translational modifications. The basic limitation is the lack of consensus on the collection of the fluid, which differs between studies.

The protein content of endometrial fluid was first studied in 1998 by Beier and Beier-Hellwig^(57)^. In 2009, Chen et al.^(58)^ reported 196 different proteins secreted from the endometrium during the mid-proliferative phase compared with the mid-secretory phase.

Proteomic analysis of uterine secretions has been the subject of much research^(49,52,54)^. In summary, the information gathered from these studies shows us that the main functions of the proteins found in uterine secretions are especially related with cytoskeleton regulation, cellular adhesion, protein folding, and signal transduction.

## LIPIDOMIC ANALYSIS OF THE ENDOMETRIUM

Compared with genomic and proteomic analysis, lipidomic analysis has thus far been the least used method among all the research on the endometrium^(59)^. On the other hand, we know that the endometrium has a very important lipid component for reproduction. Triglycerides and eicosanoids can be counted among the lipid mediators secreted from the endometrium. From the eicosanoid family, prostaglandins, thromboxanes, leukotrienes, endocannabinoids and sphingolipids have been observed to play a role in reproduction^(60,61,62,63,64)^. Among them, endocannabinoids especially, lysophosphatidic acid (LPA) and prostaglandins (PG) were the most studied agents^(65)^. The consistency of results of research conducted on different animal models supports the hypothesis that lipids could be critical for implantation.

Two basic endocannabinoids were found to play a role in implantation in rodents: anandamide N-arachidonoyl ethanolamine (AEA) and 2-arachidonoylglycerol. Abnormal levels of these two lipids lead to the postponing of implantation and a decrease in pregnancy outcomes^(66^). The basic enzyme that degrades endocannabinoids is fatty acid amide hydrolase (FAAH) and it was understood that this enzyme was associated with high pregnancy rates after IVF and embryo transfer (ET)^(65)^. It is reported that low FAAH levels leads to an increase in AEA and subsequent decrease in uterine receptivity^(67)^.

Lysophosphatidic acid is a water-soluble phospholipid. It is a signal transduction molecule that has many functions in different organs. In a study conducted in mice, LPA was shown to be necessary for normal embryo size by stimulating its receptor LPA3,^(61)^ which is a positive factor for implantation. According to a recent study, lysophosphatidic acid regulates the endometrium for implantation during the receptive period by controlling levels of endocannabinoid and prostaglandin mediators via its receptor LPA3^(64,65)^.

Prostaglandins (PG) are the final products of arachidonic acid metabolism. Phospholipids present in membranes turn into PG with phospholipase A2 (PLA2) and COX-2. With these enzymes, PG levels in the lumen and stroma increase. Animal studies reported interesting results about the effects of PG and COX enzymes on reproduction. For example, it was observed in mice that the PG increase is limited in the area where the blastocyst implants^(68)^. In that study, pregnancy in mice was totally ‘prevented’ with the inhibition of COX-1 and COX-2, thus showing the importance of prostaglandins during pregnancy^(68)^. The prevention of pregnancy was restored partially with the exogenous application of PGI2 and PGE2. In this case, prostaglandins must play an important role in embryo implantation in mice. In another study, PGE2 was found increased in the implantation area, whereas PGF2-α was mostly found between implantation areas^(69)^. However, in 2006, Cong et al.^(70)^ reported that when the implantation period was compared with other periods of the menstrual cycle, PGF2-α was not increased; the highest increase was seen in PGI2 followed by PGE2. PGI2 and PGE2 is known to increase the vascular permeability, which is necessary for ovulation and the start of implantation^(71)^. However, in one study conducted on mice, the mediator that increased vascular permeability in the implantation area was found to be PGE2, not PGI2^(59)^. PGE2 was found to play a positive role in implantation in different kinds animals^(60,72,73)^. In 2013, Vilella et al.^(74)^ collected 51 endometrial fluid samples from women and observed that PGE2 and PGF2α levels were significantly increased during the implantation window.

In a recent study, a relation between defective endometrial prostaglandin synthesis and repeated implantation failure was established in women undergoing IVF treatment^(75)^. This study showed that prostaglandins were important mediators at early-stage pregnancy.

## CONCLUSION

Endometrial receptivity is a crucial process during embryo implantation. The common result of all the genomic analyses mentioned in this review is that endometrial receptivity is a complex process that involves countless genes. Some studies report that some genes are continuously expressed in the endometrium and modifications in these genes can help us to better understand endometrial receptivity.

Proteomic and lipidomic research has started to find its place in reproductive medicine. It will take time for the results of research to become a specialized tool for diagnosis and treatment. At the same time, even potential suppositions are exciting. For example, drugs that women use during IVF treatment might change the molecular composition of endometrial tissue and secretions by altering the physiology of the endometrium. With the help of proteomic and lipidomic analysis, objective treatment protocols developed according to the patient’s personal features can be identified. In this context, genomic, proteomic, and lipidomic analysis will probably have to be used together.

In conclusion, the genomic, proteomic, and lipidomic analysis of endometrial tissues and secretions can provide investigators with important information about how genes, protein, and lipid mediators work and how they can enable endometrial receptivity ready for embryo implantation. Thus, effective treatment protocols can be developed in order to increase the basic aim of IVF strategies, the pregnancy rate. The subsequent step is testing the endometrium in terms of receptivity and guiding modifications of ovarian stimulation and luteal phase support protocols.
